# Consumption or Not

**Published:** 1867-04

**Authors:** 


### HALL'S JOURNAL OF HEALTH.

Our Legitimate Scope is almost boundless: for whatever begets pleasurable and harmless feelings, promotes Health; and whatever induces disagreeable sensations, engenders Disease.

WE AIM TO SHOW HOW DISEASE MAY BE AVOIDED, AND THAT IT IS BEST, WHEN SICKNESS COMES, TO TAKE NO MEDICINE WITHOUT CONSULTING A PHYSICIAN.

Vol. XIV.] APRIL, 1867. [No. 4.

### CONSUMPTION OR NOT.

The following letter received within a week, from a medical gentleman, who has written much, and wisely and well, is corroborative in part, of what has appeared in these pages within the last three months; and if it shall aid in persuading the reading public to give their confidence and patronage to men who are known of all, and who have a reputation to sustain in giving true opinions as to health and disease, a great good will be gained. It would seem that any man of common sense ought to have learned before he was thirty, that the most positive people are the most ignorant, both as to matters of law and medicine. The lawyer who will tell you that you will certainly gain your case, wants a fee badly, and so does the physician. Medical men of eminence and skill and long experience can seldom be induced to say more, in reference to any ordinary case of sickness. "I think you can get well." "There is encouragement to believe that you will recover." Inexperience only, is positive. It is the charlatin who lies remorselessly, and he lives by it, and yet in four cases out of five, the patient will intrust his health and life to the keeping of the man who is most profuse and brazen and self-confident in his promises of amendment. There are cases occurring every day, in which the experienced physician can say with certainty, it is not this disease, it is not that. It is this, it is that, and cannot be otherwise, it is as to the results of treatment that all should speak with great hesitation; first, because it cannot be told whether the patient has moral courage and force of character enough to carry out the instructions. Second, it cannot be told, until the trial is made, in some cases, whether there is vitality enough to recover from the disease even after it is cured; very many persons, for example, have died, after they were cured of the cholera. Fevers are often subdued, but at so late a date in the progress of the disease, that there was not enough recuperative power left to rally. Third, a medicine may do all that is wished and all that is needed for nine times, yes, for ninety-nine times, and yet at the hundredth it will fail, because of some peculiarity in the constitution of the patient called "Idiosynchrasy," by which it fails to have any power of action on any bodily organism. Tartar Emetic will not vomit everybody. The sea will not sicken all who sail upon it.

"I little thought ten years ago," wrote a gentleman of high culture and social position, "that I would ever have any interest in a book of yours, which I saw announced in the public papers, and turned over its leaves with almost indifference, but now alas things have changed, and I have greedily read every line you have written, for I fear consumption is upon me." Hence the reader is counselled to preserve with great care the January, February and March numbers for 1867, now bound in one cover, and sent, post-paid, for fifty cents, as they give a very full and truthful view of the principal diseases of the throat and lungs, and how to distinguish them, for either in your own person or in that of some member of your family, or your friends, you will certainly feel the need of the information desired. Meanwhile, the letter referred to, is appended.

BOSTON, MASS., Feb. 26, 1867.

W. W. HALL, M.D.:

DEAR SIR—Though I have never seen you, yet, for many years, I have seen and known *of* you and yours, on *health.* I have just read your article, entitled, "Vital Capacity," in the number of your Journal of Health, for March; and, you will pardon me for troubling you with a few words on the same subject. Like yourself, for a quarter of a century, I have given special attention to diseases of the *Throat* and *Lungs,*

first led to this practice by some family traits. During this period, many cases have come under my observation, and I have seen so much of what you have so graphically and justly described, that I cannot refrain from confirming your statements by relating some of them to you.

"—it is so much less troublesome to speak the truth ; is such a relief to be free from the incubus of a falsehood," &c. I like all you have said in this connection. Twenty years ago this winter, I had a young lady patient, in consumption. There could not be a doubt in her case. I stated, frankly, to her parents that her case was a *critical* one ; and, if they wished for further counsel, I should like to have them secure it. They had full confidence in me and said, they wanted none other. I then told them, "they must not feel disappointed if their child did not live. In a word, that I thought she could not." At my next visit, the father said, "though we have full confidence in you, yet we have heard of a young woman, in another part of this town, who was very much as our daughter is, and she has been cured by Dr.—— of Boston. Would you have any objection to his seeing her?" By no means, said I, he is a member of the Massachusetts Medical Society ; will you ask him to see her *with* you? I will. I did so. He came, examined the case, prescribed. The mother followed him into an adjoining room and asked him "can our daughter get well?" Oh! yes, said he, she will be better soon. Thus he left her with the impression that she would recover.

We entered the car together to return to the city. I said, Dr. do you think there is any chance for that girl to recover? "Oh! I don't know," said he, "you know, we never tell the family what we think." In four weeks she died. For twenty years that mother, who still lives, with gratitude, has said, "you told us the truth."

"If a man is not consumptive, and is plainly told so, such a burthen is sometimes taken from his mind, that a new life is infused into him" &c. Words fitly spoken, Doctor, and for them you deserve the praise of all good men.

Fifteen years ago a young man belonging in the western part of this State, called on me, and asked me to examine his lungs. He said, "they had been examined three days before by a physician in New York, who had told him they were badly diseased; and in answer to the question, how long could he live, the Doctor replied not more than three months."

I examined him carefully, and told him, he had not consumption, and that I thought he would recover. Your words were verified, "new life was infused into him." He went home, and I have now in my possession a letter, written one year after this examination, in which he says, "my health is now good."

While I was in Philadelphia, a near relative came from near Boston, to stop with us, expecting to die of consumption. He came with his wife, and bed-blankets to wrap himself in, bringing his *hypophosphites* and other medicines, which I still have. I examined him and told him, he had no disease of the lungs. I found an enlarged liver, and a sympathetic cough, and considerable debility. I put him upon the old remedy of nitromuriatic acid, and never changed it, and in four months he was well, and has been so ever since,—now four years.

Thus, Dear Sir, I have verified your statements on *both* sides, and can pronounce them *true*.

One other remark of yours I have found equally true—"it is reasonable to suppose, when a physician is called upon to give his candid opinion of a case, that the family and friends would feel themselves under considerable obligations, when such an opinion was given, although unfavourable, especially when that opinion was subsequently found to have been literally correct. But this is not always the case."

This, also, I have seen verified; unaccountable as it may seem, it is too often *true*.

I pretend to no infallibility, or knowledge above that of my brother doctors. But, I can say, I have very rarely been mistaken in these cases, and I have always given my opinion frankly when asked to do so, and I do not think any physician can be justified in pursuing a different course.

In conclusion let me thank you for your views on these important points, so candidly and honestly expressed. And, also, for the great amount which you have written so well, that none could have done it better, upon the means of preserving health, you *have* your reward in an appreciating public, and the universal esteem of all your readers.

The next time I visit New York, (Deo Volente) I purpose to see your face.

In the meantime, I remain very truly yours, &c.

---

" Cacoethes Scribendi," an itch for writing, an avalanche of wordiness. Some persons in writing for a book or magazine, will spoil a double page of good paper, when the whole letter, including place, name, date and all, ought to come within twenty words; a good way to acquire succinctness, is to take the reverse of an old envelope, and compel yourself to say all you want, on the unused side of it. Time is money in New York, and editors, lawyers, doctors and clergymen are obliged to husband every moment of day light, for every day in the year. The fact is, no public man in this city, can do anything which he is not obliged to do,as a very general rule. If a physician is called to see a sick friend, he does it on the instant; but if he purposes making that friend a social visit of half an hour, the time does not come, sometimes in years, when every thing suits to make that visit; and often, very often, while he is waiting for the opportunity, he reads in the morning paper, " died yesterday," then, regrets and self-reproaches come on apace, and he exclaims, " why didn't I go and see him." Yet, notwithstanding this great busyness of those who live in large cities, they are constantly called upon by friends in the country to perform various little offices, as they seem to be to outsiders, which will take half a day's time to attend to. One wishes you to subscribe for some publication, another to purchase him a book, another to call and pay a little bill, or some other of a multitude of " trifles " in their way ; but small as they are, they consume time. If a friend writes to us to step down town and subscribe for his favorite paper or purchase him a new book, it requires a journey of seven miles to do this same, and two hours of precious daylight, and in attending to such things, our absence from our office, has frequently cost us the loss of a liberal fee. The fact is, we have been a pack-horse for others from our earliest remembrance, and have come at last to adopt the conclusion, that the better half of mankind were destined to be ridden by the triflling, lazy, loafing half.

Not long since a "relation," yes how "relations," specially the poor ones, hang on to a fellow, "like grim death to a" cullud pusson, wrote to us, requesting that we would call and get a valuable family painting, and forward it to him. Well we called, seven miles off, and it was not there, sent a letter eight hundred miles to find out where it was, then made a journey of six miles more; while we were gone, we lost a good round fee, from a man who called with a hemorrhage, of course couldn't wait, and he went elsewhere. A fortnight ago, a pretty country cousin wrote in a way perfectly undeniable, to know if we wouldn't call on Broadway, and get her dress, which had been left for some fixing. We called, of course we called, how could we help calling, when the handsomest woman we knew had given her commands; the people couldn't find the article, wanted to know if we knew it, then after waiting an interminable time, sitting on a stool, twirling it round, the little French gipsy came to ask, if the owner of the dress was a relation of ours; we didn't see the point, but promptly and succinctly answered, "Yes," then there was a pause, both parties seeming to wait for something else, but we were exhausted of all our talk, the little gipsy had such pretty curls, and such a sweet lisp and Frenchy way of talking English, we forgot all about the dress, for present beauty outvies the absent. At length we were requested to please ask our relation to step down, and perhaps she could recognise her own dress; but we had to inform our little witch that the step would be rather a longer one than a lady usually takes, as she was just a thousand miles out of town. We were then inquired of, if we wouldn't have the kindness to call next "evening." We couldn't exactly tell what was the necessity of the "evening" part of the business, married men have no business out of evenings, besides we get sleepy as soon as dark comes, and are in bed at nine o'clock; so we simply said, "will call again." In a few days we did call, to be informed of their sorrow in not having found the dress, and that if we would call in a few days we would then be informed when to call again. Now as we never like to give up what we set out for, we intend to keep on visiting the little French girl until our cousin's dress turns up, or some other dress turns up. By all of which we mean to say that we don't like to be unaccommodating, but at the same time ask our friends to consider that an editor has no spare time from one end of the year to another, and that if they want any little jobs done, the best plan would be to write to P. C. Godfrey or Henry B. Price, New York, who will do anything faithfully and promptly, from the purchase of a dollar book to a steam engine, or a Fifth avenue house, for a very moderate consideration.

